# Editorial: Mental illness and neuropsychiatry of the homeless: psychosis, personality, drug abuse, and other brain disorders

**DOI:** 10.3389/fpsyg.2024.1447883

**Published:** 2024-07-03

**Authors:** João Gama Marques, Joana Henriques-Calado, Martin M. Schumacher

**Affiliations:** ^1^Consulta de Esquizofrenia Resistente, Hospital Júlio de Matos (HJM), Unidade Local de Saúde de São José (ULSSJ), Centro Clínico Académico de Lisboa (CCAL), Lisboa, Portugal; ^2^Clínica Universitária de Psiquiatria e Psicologia Médica (CUPPM), Faculdade de Medicina Universidade de Lisboa (FMUL), Centro Académico de Medicina de Lisboa (CAML), Lisboa, Portugal; ^3^Homeless Outreach Psychiatric Engagement for Lisboa (HOPE 4 Lisboa), Santé Mentale et Exclusion Sociale (SMES), Lisboa, Portugal; ^4^Centro de Investigação em Ciência Psicológica (CICPSI), Faculdade de Psicologia, Universidade de Lisboa (FPUL), Lisboa, Portugal; ^5^Faculdade de Psicologia, Universidade de Lisboa (FPUL), Lisboa, Portugal; ^6^Independent Researcher, Sissach, Switzerland

**Keywords:** psychiatry, neuropsychiatry, psychology, neuropsychology, homeless

The ailments of persons experiencing homelessness have been studied by clinicians, but also academics, from different backgrounds for many decades. A rapid search on PubMed, using the word homeless, revealed its first use by in 1888 (Henderson, 1888). Although the author might not have been the first one to use the concept, it seems that it was the first time the word appeared in this important database. Since then, many other works focused in the homeless. Today we celebrate three of those:

## One hundred years of “the hobo: the sociology of the homeless man” by Nels Anderson

In November 2023 we celebrated the 100th anniversary of the seminal work “*The hobo: the sociology off the homeless man*” by Nels Anderson, a sociologist, that in 1923, Chicago, United States of America, distinguished hobos, tramps, bums and home guards (Anderson, 1923). In the following decades, countless authors realized the complexity of persons experiencing homelessness, exploring other concepts, such as vagrants (Kirchesch, 1950), skid rows (Myerson, 1953), runaways (Robins and O'Neal, 1959), urban nomads (Gropper, 1967), drifters (Bandler, 1967), squatters (Pataki-Schweizer, 1978), street people (Jones, 1983), throwaway people (Curtin, 1986), street youth (Côté, 1989), space cases (Fischer, 1992), gutter punks (Goetz, 2000), squeegees (Dachner and Tarasuk, 2002), etc.

## Thirty years of “*Santé Mentale et Exclusion Sociale*” by Luigi Leonori

In December 2022 we celebrated, the 30th anniversary of the European organization *Santé Mentale et Exclusion Sociale (SMES)*, created by Luigi Leonori, a professor of Psychology, in 1992, Rome, Italy. He and his colleagues were worried about the social exclusion (“*Exclusion Sociale”*), of persons with mental health (“*Santé Mentale”*) problems, experiencing homelessness (http://www.smes-europa.org/). Not only in Europe, persons experiencing homelessness have been labeled with a considerable number of different designations: *pixote* (Brazil), *gamino* (Colombia), *itinérants* (Canada), *clochard* (France), *puliukko* (Finland), *sans-abri* (France), *pennebruder* (Germany), *barboni* (Italy), *tunawisma* (Indonesia), *furosha* (Japan), *sin techo* (Mexico), *khate* (Nepal), *desamparado* (Peru), *sem-abrigo* (Portugal), or *BOMZI*, the acronym for *Bez Opredilyonogo Mesta Zhitelstva* (Russia), etc (Glasser, 1994).

## Twenty years of “*Sem-Amor Sem-Abrigo”* by António Bento and Elias Barreto

In September 2022 we celebrated the 20th anniversary of “*Sem-Amor Sem-Abrigo”*, a book published in 2002, in Lisboa, Portugal, by the recently deceased António Bento (Gama Marques, 2024), a psychiatrist, and Elias Barreto, a psychologist. At the time, the authors interviewed a small sample of homeless men and found not a single case of secure attachment style, leading them to propose the loveless (*sem-amor*) hypothesis among the homeless (*sem-abrigo*) (Bento and Barreto, 2002). Interestingly, all around the world, many other authors also looked on persons experiencing homelessness, as human beings lacking other important things, besides love: jobless (Miller et al., 1970), rootless (Holden, 1975), houseless (Bailey, 1977), supportless (Lipton and Sabatini, 1984), defenseless (Farr, 1985), restless (McLaughlin and Pepper, 1990), familyless (Liebow, 1993), roofless (Newton et al., 1994), nameless (Gama Marques and Bento, 2020a,b), healthless (Yen et al., 2009), shelterless (Burton et al., 2020), etc.

Here in Portugal, we have been trying to follow charismatic leaders, inspired by true homelessness champions who brought people together and held on to a vision (Pannel and Parry, 1999), such as António Bento and Luigi Leonori in Southwestern Europe, or Mitch Snyder (Snyder and Hombs, 1986) and Edwin Fuller Torrey (Fuller Torrey, 1988) in Northeastern America.

We published papers revisiting theoretical concepts such as marontology, comorbidity (Gama Marques and Bento, 2020a,b), super-difficult patients (Gama Marques, 2021), mortification and shelterization (Gama Marques, 2022a,b). We did reviews on homelessness and epilepsy (Pontes Silva and Gama Marques, 2023), schizoaffective psychoses (Spranger Forte et al., 2023), and attachment disorders (Neves Horácio et al., 2023). And we spread case reports of homeless patients with conditions such as haltlose personality disorder (Gama Marques, 2019), treatment resistant schizophrenia, organic psychosis, pellagra, Capgras delusion (Gama Marques, 2022a,b), Huntington chorea, John Doe and Diogenes syndromes (Gama Marques, 2023).

**Figure 1:**
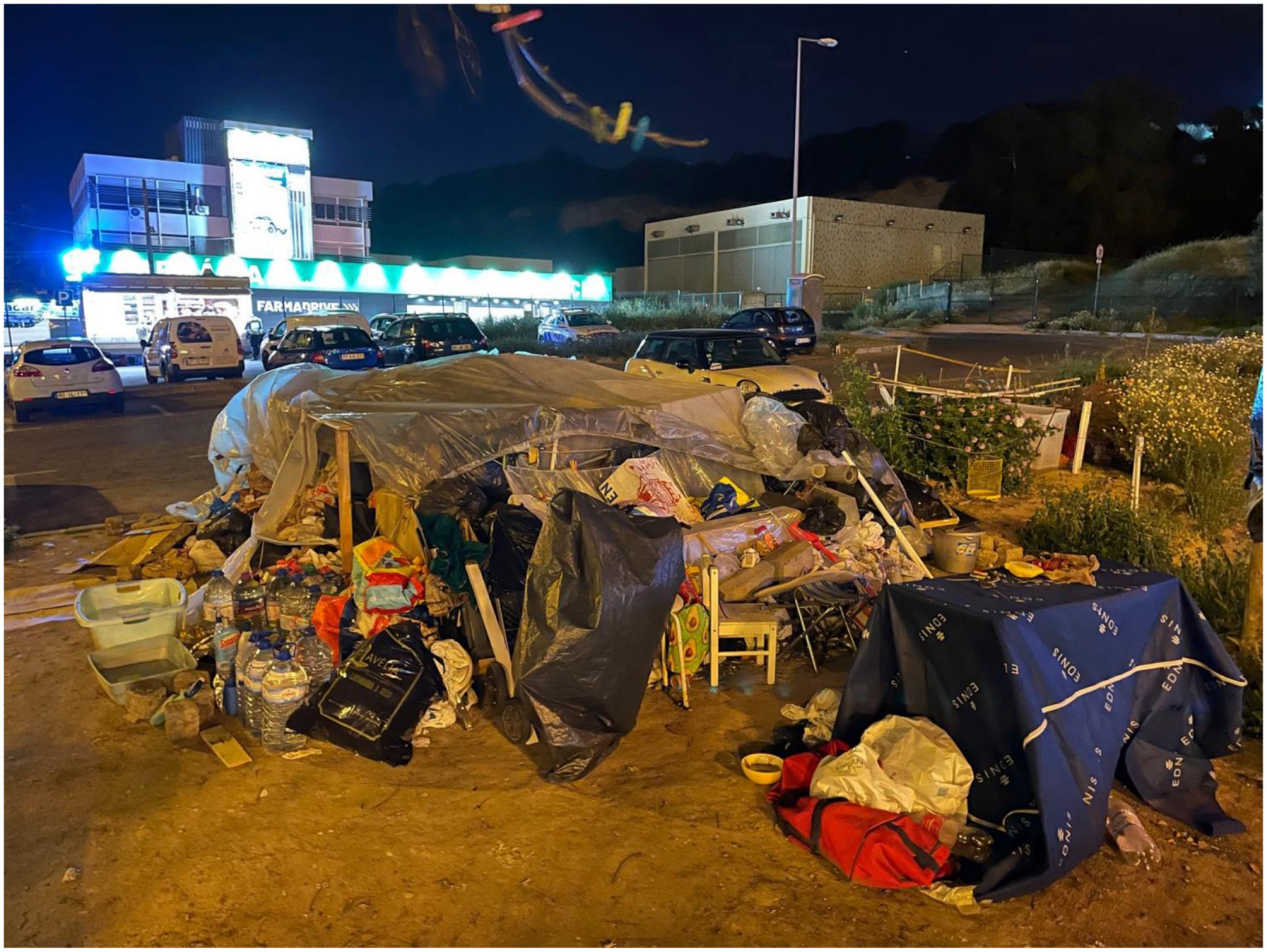
The sleeping ground of two of our psychotic patients, a middle age couple sharing, for more than a decade, a grandiose *folie a deux* with indescribable ruin and misery.

We have been doing interstitial or street psychiatry, while leading the Homeless Outreach Psychiatric Engagement for Lisboa (HOPE 4 Lisboa) (Monteiro Fernandes et al., 2022; Gama Marques et al., 2023). Figure 1 represents just an example of our work: the sleeping ground of two of our psychotic patients, a middle age couple sharing, for more than a decade, a grandiose *folie a deux* with indescribable ruin and misery.

The present Research Topic on Mental Illness and Neuropsychiatry of the Homeless: Psychosis, Personality, Drug Abuse, and Other Brain Disorders, compiles ten articles from both sides of the Atlantic Ocean, on five different Frontiers journals.

**Frontiers in Human Neuroscience**: an Original Research manuscript, by Rangu et al. (Oklahoma), describes a relation between head concussions and medication non-adherence; and a Brief Research Report, by Pluck (Thailand) raises a pertinent question: is executive dysfunction among the homeless a true impairment or just another case of frontal lobology?

**Frontiers in Artificial Intelligence:** an Original Research article, by Chapman et al. (Utah), assesses the longitudinal housing status, of patients, using electronic health record data.

**Frontiers in Psychiatry**: one Opinion by Bravo et al. (Portugal); and one Brief Research Report, by Herrera-Imbroda et al. (Spain), both regarding the problem of readmissions in the homeless population; one Mini Review, by Henriques-Calado and Gama Marques (Portugal) dedicated to personality disorders; and a Systematic Review, by Hird et al. (New Haven), looking at the approaches to improve medication adherence in the homeless population.

**Frontiers in Psychology**: a Community Case Study by Gabrielian et al. (California), on the engagement of stakeholders in a homeless veterans' program; and one Original Research article, by Oliveira Azevedo et al. (Portugal), dedicated to a harm reduction intervention with homeless people struggling with alcoholism.

**Frontiers in Public Health**: one Brief Research Report, by Catthoor et al. (Belgium) looking at the housing problems in admitted psychiatric patients.

We regret not having more articles published in this topic. Nevertheless, we hope our Research Topic' articles will stimulate future discussion regarding persons living and dying with psychiatric disorders and neurologic diseases while experiencing homelessness.

## Author contributions

JG: Conceptualization, Investigation, Writing – original draft, Writing – review & editing. JH-C: Supervision, Validation, Writing – review & editing. MS: Supervision, Validation, Writing – review & editing.

## In Memoriam

Dr. António Bento, 1954–2023 (Lisboa, Portugal, European Union).
